# Synthesis, Anti-Tumor Activity and Apoptosis-Inducing Effect of Novel Dimeric Keggin-Type Phosphotungstate

**DOI:** 10.3389/fphar.2020.632838

**Published:** 2021-01-27

**Authors:** Yingxue Xue, Yifei Yin, He Li, Mingyu Chi, Jiaxin Guo, Guihua Cui, Wenliang Li

**Affiliations:** ^1^ School of Pharmacy, Jilin Medical University, Jilin, China; ^2^ Research and Development Department, NCPC Hebei Lexin Pharmaceutical Co., Ltd., Hebei, China; ^3^ Jilin Collaborative Innovation Center for Antibody Engineering, Jilin Medical University, Jilin, China

**Keywords:** anti-tumor, apoptosis, dimeric, Keggin-type, phosphotungstate

## Abstract

A dimeric Keggin-type phosphotungstate (ODA)_10_[(PW_11_FeO_39_)_2_O]·9H_2_O (abbreviated as ODA_10_[(PW_11_Fe)_2_], ODA = octadecyltrimethylammonium bromide) was synthesized and investigated comprehensively its antitumor activity on MCF-7 and A549 cells. The dimeric structure and amorphous morphology were characterized by FT-IR, UV-vis-DRS, SEM and XRD. The *in vitro* MTT assay of ODA_10_[(PW_11_Fe)_2_] showed anticancer activity on MCF-7 and A549 cells in a dose- and time-dependent manner, and the IC_50_ values for MCF-7 and A549 cells at 48 h were 5.83 μg/ml and 3.23 μg/ml, respectively. The images of the ODA_10_[(PW_11_Fe)_2_]-treated cells observed by inverted biological microscope exhibited the characteristic morphology of apoptosis. Flow cytometric analysis showed cell apoptosis and cycle arrested at S phase induced by ODA_10_[(PW_11_Fe)_2_]. The above results illuminated the main mechanism of the antitumor action of ODA_10_[(PW_11_Fe)_2_] on MCF-7 and A549 cells, indicating that this dimeric phosphotungstate is a promising anticancer drug.

## Introduction

With the increasing morbidity and mortality, cancer has become a major killer that leads to health- and life-threatening for humans all over the world (Sun et al., 2019). At present, the main methods for the treatment of cancer are surgery, drug therapy, radiation therapy and cryotherapy. Among them, chemotherapy is also an effective method for cancer treatment (Xiao et al., 2019; Chen et al., 2020; Liu et al., 2020). Many chemotherapeutic agents, such as cisplatin (Weiss and Christian, 1993; Chen et al., 2018), fluorouracil (Arkenau et al., 2003) and capecitabine (Ssif et al., 2008), etc., have shown the potential for alleviating symptoms and curing the cancer (Cao et al., 2020; Geraldi, 2020; Zhao et al., 2020). However, most chemotherapeutic drugs possess inherent disadvantages, such as poor selectivity, severe side effect, low efficiency and drug resistance in cancer cells (Carr et al., 2008; Jonckheere et al., 2014; Hamis et al., 2018). Therefore, it is necessary to design drugs with high efficiency and low toxicity.

Polyoxometalates (abbreviated as POMs) are a series of transition metal oxygen anion clusters, which are mainly composed of molybdenum (Mo^Ⅵ^), tungsten (W^Ⅵ^), vanadium (V^Ⅴ^), niobium (Nd^Ⅴ^), and tantalum (Ta^Ⅴ^) in their highest oxidation state bridged by oxygen atoms (Rhule et al., 1998; Wang et al., 2003). Intriguingly, many other elements can be incorporated into the framework of POMs, leading to the diversity in structures and properties (Dianat et al., 2015), such as redox potential, polarity, thermal stability and electronic properties, etc., making them attractive for application in the fields of catalysis (Mizuno et al., 2005; Hill, 2007), electrochemistry (Goura et al., 2020), material science (Du et al., 2010) and medicine (Muller et al., 1998). Jasmin et al. (1974) firstly reported the antivirus activity of (NH_4_)_17_Na[NaSb_9_W_21_O_86_] (HPA-23) against sarcoma virus. Since then, more POMs have been found to exhibit antitumor (Boulmier et al., 2017; Bijelic et al., 2019), antibacterial (Ma et al., 2020), antivirus (Qi et al., 2013), and antidiabetic activities (Liu W. J. et al., 2016). It is reported that POMs are significant antitumor drug candidates with high efficiency and low toxicity for curing most types of cancers, such as pancreatic cancer, breast cancer, leukemia, colon cancer, ovarian cancer and so on (Li et al., 2017; Hu et al., 2019).

The unique advantage of POMs over current drugs lies in the fact that the molecular structure and physiochemical properties of POMs are tunable and can be easily synthesized from readily available precursors in a few synthetic steps (Judd et al., 2001; Müller et al., 2006). POMs can be surface modified with synthetic organic compounds or natural molecules to effectively improve the biological activity *in vitro* and/or *in vivo* (Sun et al., 2016; Van Rompuy and Parac-Vogt, 2019). Electrostatic interaction, as a method of surface modification of POMs, combining organic countercations (such as quaternary ammonium salts) with POMs anions together, which makes the formed POMs take advantage of the synergistic effect and enhance the antitumor activity (Yu et al., 2014; Qu et al., 2017; Cheng et al., 2018). Quaternary ammonium salts are widely used as an antibacterial agent against a variety of bacteria, fungi and virus (Diz et al., 2001), which is based on the diversity in properties of low-molecular weight, outstanding cell membrane penetration, extended residence time, low toxicity, good biological activity and environmental stability (Dizman et al., 2006).

On the other hand, Keggin-type POMs were gaining increased interest as antitumor and antivirus agents due to the simple structure, small size and being easily synthesized (Shigeta et al., 2003; Zheng et al., 2009; Liu X. et al., 2016), such as K_6_H[CoW_11_O_39_CpM]·nH_2_O (M = Zr, Ti, Fe, Cp = *η* ^5^-C_5_H_5_), Ag_3_[PW_12_O_40_], Ag_6_[SiW_10_V_2_O_40_] and Ag_4_[SiW_12_O_40_], all exhibiting inhibitory effect on tumor cells and sporotrichosis (Dianat et al., 2013; Mathias et al., 2020), respectively. But the work involving both the synthesis and antitumor effect of dimeric Keggin-type POMs are seldomly reported. Although the synthesis of dimeric [N(CH_3_)_4_]_10_[(PW_11_FeO_39_)_2_O]·12H_2_O (Pichon et al., 2008) and [Bmim]_10_[(PW_11_FeO_39_)_2_O]·0.5H_2_O (Santos et al., 2012) were reported, the further studies on the antitumor efficacy and mechanism of dimeric Keggin-type phosphotungstate with quaternary ammonium cation are not very frequent.

The exact mechanism of cancer cells death induced by POMs is still unknown, but it is reported that the antitumor activity of POMs correlates with their biological activities, including immunomodulatory (Sun et al., 2010), apoptotic (Cao et al., 2017) and inhibition effects toward enzymes (Prudent et al., 2008). Due to the lack of a comprehensive research on the biological mechanism of POMs, compared to much more common organic drugs, POMs as inorganic drugs are still rarely applied in pharmacy field (Guo and Sadler, 1999). So, considerable attention has been paid to the cellular and molecular mechanisms between tumor cells and POMs.

In the present work, we have chosen the quaternary ammonium salt with relatively long alkyl chain of octadecyltrimethylammonium bromide (ODAB) as organic counteraction, which is expected to exhibit better biocompatibility and higher cell membrane penetration, because the biological activity of quaternary ammonium salts correlates with their molecular structure and the length of the carbon chain. The longer alkyl chain of the compounds contributes to higher antibacterial activity of theirs (Abel et al., 2002). Herein, it is firstly reported that a dimeric Keggin-type polyoxometalate (ODA)_10_[(PW_11_FeO_39_)_2_O]·9H_2_O (ODA_10_[(PW_11_Fe)_2_]) was synthesized based on electrostatic interaction between octadecyltrimethylammonium cation and [(PW_11_FeO_39_)_2_O]^10−^ anion. The structure and morphological feature of ODA_10_[(PW_11_Fe)_2_] were characterized. The *in vitro* antitumor activity of ODA_10_[(PW_11_Fe)_2_] on MCF-7 and A549 cells was investigated. And the morphological changes and cell density of MCF-7 and A549 cells induced by ODA_10_[(PW_11_Fe)_2_] were detected by inverted biological microscopy. Furthermore, cell apoptosis and cell cycle distribution were analyzed.

## Materials and Methods

### Materials

Sodium tungstate dihydrate (Na_2_WO_4_·2H_2_O), Sodium phosphate dibasic (Na_2_HPO_4_), Iron nitrate nonahydrate (Fe(NO_3_)_3_·9H_2_O), Sodium bicarbonate (NaHCO_3_) and octadecyltrimethylammonium bromide (ODAB) were acquired from Sinopharm Chemical Reagent Co. Ltd., China. Cisplatin and carboplatin were purchased from Nanjing Jingzhu Bio-technology Co., Ltd. (Nanjing, China). Trypsin, phosphate buffer saline (PBS), dimethyl sulfoxide (DMSO) and 3-[4,5-dimethylthiazol-2-yl]-2,5-diphenyltetrazolium bromide (MTT) were obtained from Sigma (St. Louis, MO, United States). Fetal bovine serum (FBS), RPMI-1640 Medium and penicillin-streptomycin were purchased from Gibco BRL (Grand Island, NY, United States). Annexin V-FITC apoptosis detection kit and cell cycle kit were obtained from BD Biosciences (San Jose, CA, United States). All chemicals and solvents were used as received from commercial sources without further purification. (TEA)_10_[(PW_11_FeO_39_)_2_O]·3H_2_O and (TMA)_10_[(PW_11_FeO_39_)_2_O]·4H_2_O (TEA = tetraethyl ammonium bromide, TMA = tetramethyl ammonium bromide) were prepared according to the literature (Pichon et al., 2008), and the characteristic data were consistent with previously published values (Pichon et al., 2008; Santos et al., 2012).

### General Measurements

The content of P, W, Fe in the phosphotungstate was performed on an ICP-OES Plasma Spec (Thermo iCAP 6000), and the elemental analysis of C, H, N was tested on a CHN elemental analyzer (Perkin-Elmer 2400). Thermogravimetric analysis (TGA) was measured on a Shimadzu DTG-60 instrument using N_2_, with a heating rate of 6°C min^−1^. Fourier Transform infrared (FT-IR) spectroscopy was tested by Nicolet-Impact 400 spectrometer using KBr disk. The UV–vis diffuse reflectance spectra (UV-vis DRS) were performed on a UV-Vis-NIR spectrometer (Agilent Technologies Cary Series) using BaSO_4_ as reference. X-ray diffraction (XRD) data were tested by a SHIMADZU XRD-6000 X-ray diffractometer with Cu Kα radiation (*λ* = 0.1548 nm). The morphology of the phosphotungstate was analyzed by scanning electron microscope (SEM) performed on a JSM-6360LV microscope. Particle size distribution was obtained by dynamic light scattering (DLS) using a Mastersizer 2000 laser particle size analyzer.

### Synthesis of (ODA)_10_[(PW_11_FeO_39_)_2_O]·9H_2_O (ODA_10_[(PW_11_Fe)_2_])

Na_2_WO_4_·2H_2_O (3.3 g) was dissolved in 20 ml of distilled H_2_O in a flask. Then Na_2_HPO_4_ powder (0.13 g) was added to the solution in a molar proportion (Na_2_WO_4_·2H_2_O: Na_2_HPO_4_) of 11:1. After 1 h of stirring at 80–90°C, conc. HNO_3_ was added drop by drop to make the pH of the solution be 4.8. Then, Fe(NO_3_)_3_·9H_2_O (0.49 g, 1.2 mmol) was added and the pH was adjusted to 4.5 by adding NaHCO_3_ (1 M) drop by drop. 1.8 g of octadecyltrimethylammonium bromide (ODAB) was added, causing yellow precipitates produced, which were then filtrated, washed twice with distilled water and dried under vacuum. Anal. (%): calcd for (ODA)_10_[(PW_11_FeO_39_)_2_O]·9H_2_O: P, 0.71; W, 46.14; Fe, 1.28; C, 28.76; H, 5.45; N, 1.60; H_2_O, 1.85; found: P, 0.78; W, 48.16; Fe, 1.33; C, 29.21; H, 5.49; N, 1.65; H_2_O, 1.91.

### Cell Lines and Cell Culture Conditions

Human breast cancer cells MCF-7 and human non-small cell lung cancer cells A549 (ATCC) were incubated in RPMI 1640 medium with penicillin (100 U/ml), streptomycin (100 μg/ml) and FBS (10%) at 37°C with 5% CO_2_ in an incubator.

### Cell Viability Studies

The anti-proliferation effect of the phosphotungstate was detected by the MTT assay. The stock solution of ODA_10_[(PW_11_Fe)_2_] with the concentration of 1 mg/ml was prepared in DMSO, and then sterilized by the methyl cellulose ester filter membrane with pore size 0.22 μm. At last, the stock solution was diluted by RPMI 1640 under sterile condition.

Cells were seeded into a 96-well plate at a density of 1 × 10^4^ cells/well. 100 μl/well of RPMI 1640 medium was added to each well to incubate the cells for 24 h, and then the medium was replaced by various concentrations of ODA_10_[(PW_11_Fe)_2_] (1, 3, 6, 12, 24 μg/ml). Each concentration has four duplicate samples. After 6, 12, 24 and 48 h, 20 μl of MTT (5 mg/ml) was added to each well, and then the plate continued to be incubated for 4 h in the incubator. Next, the formed formazan crystals were dissolved in 150 µl DMSO after removing the MTT medium. Finally, the absorbance was measured at a wavelength of 490 nm by an automatic microplate reader.

### Morphological Observation

To observe whether the density and morphology of the tumor cells induced by the phosphotungstate changed or not, cells were seeded into a 6-well plate at a density of 2 × 10^5^ cells/well for 24 h at 37°C, and then treated with different concentration of ODA_10_[(PW_11_Fe)_2_] (1–24 μg/ml). After 24 h, the cellular density and morphology were observed by the inverted biological microscopy (XDS1C, Shanghai Wanheng Precision Instrument Co. Ltd., China).

### Flow Cytometry Analysis of Cell Apoptosis

Cells were seeded into a six-well plate (2 × 10^5^ cells/well) for 24 h, and then exposed to different concentration of ODA_10_[(PW_11_Fe)_2_] (1–24 μg/ml). After 24 h, the cells were collected, washed thrice with cold PBS and then centrifugated. After discarding the supernatant, the cells were resuspended in Annexin-V-FITC/PI solution and remained in the dark for 15 min at room temperature. The cell apoptosis was determined on a FAC Scanto™ flow cytometer (Becton Dickinson, United States).

### Flow Cytometry Analysis of Cell Cycle Distribution

Cells were seeded into a six-well plate (10^6^ cells/well) for 24 h at 37°C, and then exposed to different concentration of ODA_10_[(PW_11_Fe)_2_] (1–24 μg/ml). After 24 h, the cells were collected using trypsin, centrifugated and then washed with PBS for two times. After being fixed by ice-cold 70% ethanol at 4°C overnight, the cells continued to be washed with cold PBS and resuspended in propidium iodide (PI) staining solution in the dark at 37°C for 30 min. Finally, the cell cycle was determined on a FAC Scanto™ flow cytometer (Becton Dickinson, United States).

## Results and Discussion

### Synthesis and Characterization of the Dimeric Keggin-Type Phosphotungstate

**FIGURE 1:**
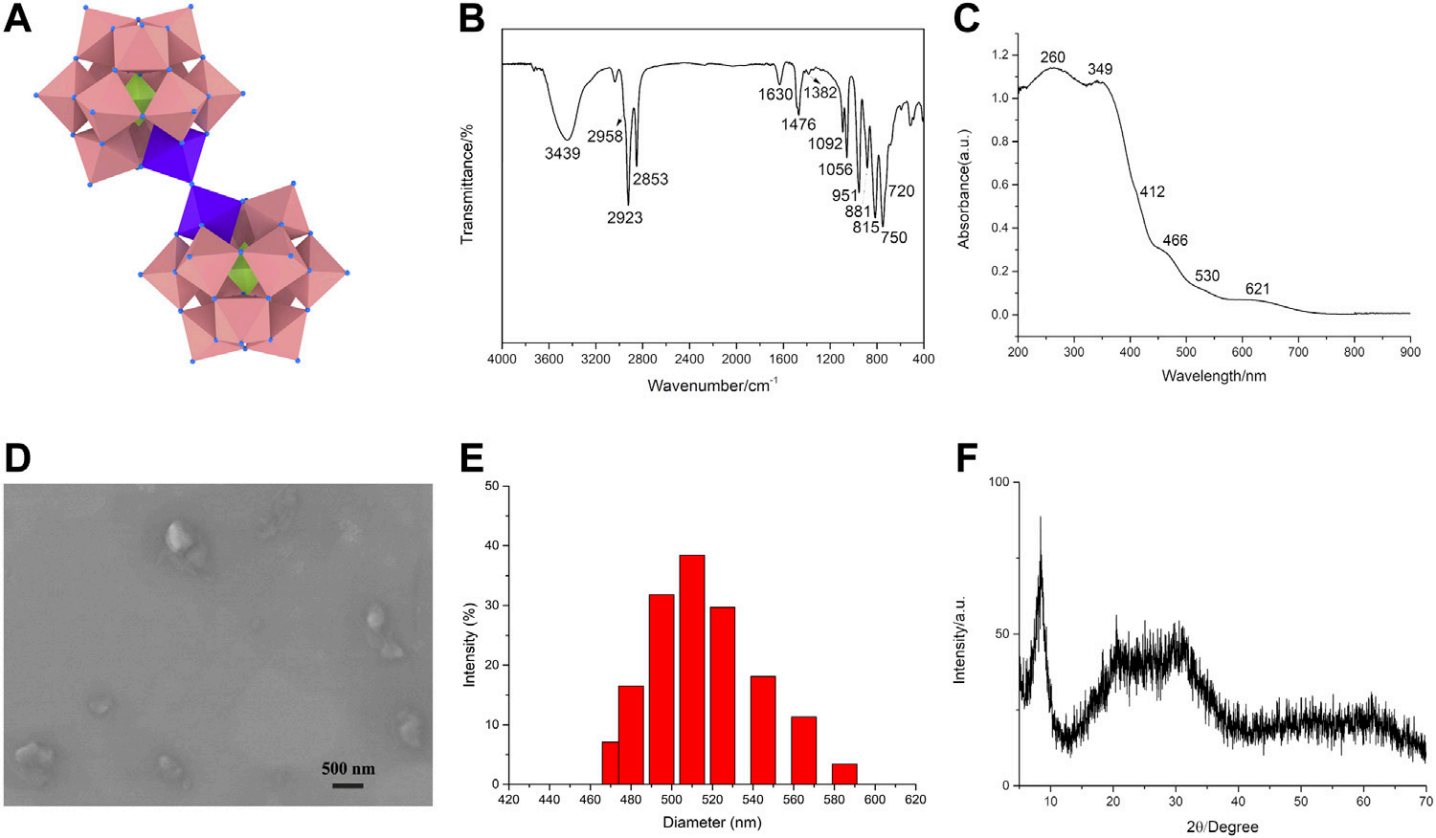
The structure and characterization of the dimeric Keggin-type phosphotungstate. The structure of ODA_10_[(PW_11_Fe)_2_] **(A)**. IR spectrum **(B)**, UV-vis DRS **(C)**, SEM image **(D)**, DLS characterization **(E)**, and XRD pattern **(F)** of ODA_10_[(PW_11_Fe)_2_].

The adjustment of pH is vital to the synthesis of the dimeric oxo-bridged [(PW_11_FeO_39_)_2_O]^10-^ anion, which usually exists at pH = 3–5 (Liu K. et al., 2016). The dimeric phosphotungstate ODA_10_[(PW_11_Fe)_2_] consists of two phosphotungstate units PW_11_Fe^Ⅲ^O_39_ which are linked by Fe-O-Fe bond, the structure is shown in Figure 1A.

FT-IR spectra provided clear evidence for the successful preparation of the dimeric Keggin-type phosphotungstate ODA_10_[(PW_11_Fe)_2_] (Figure 1B). IR spectrum of ODA_10_[(PW_11_Fe)_2_] showed characteristic peaks of Keggin-type polyoxometalates (Kuznetsova et al., 1996), which appeared at 1,092, 1,056, 951, 881, 815 cm^−1^. The peaks at 1,092, 1,056 cm^−1^ were assigned to asymmetric P-O_a_ stretching band. The peak at 951 cm^−1^ corresponded to ν_as_ (W = O_d_) vibration. The peaks attributed to ν_as_ (W-O_b_-W) and ν_as_ (W-O_c_-W) vibrations appeared at 881 and 815 cm^−1^, respectively. In addition, the asymmetric Fe-O-Fe stretching vibration of dimeric phosphotungstates was observed at 750 cm^−1^ with a shoulder around 720 cm^−1^ (Kuznetsova et al., 1996; Kuznetsova et al., 1997), which illuminated the dimeric structure of ODA_10_[(PW_11_Fe)_2_]. The IR peaks ascribed to the -CH_2_ asymmetric and symmetric stretching bands appeared at 2,923 and 2,853 cm^−1^, showing the existence of quaternary ammonium in ODA_10_[(PW_11_Fe)_2_]. The bands attributed to the -CH_3_ asymmetric stretching and scissoring modes were observed at 2,958 and 1,382 cm^−1^, respectively. The peak at 1,476 cm^−1^ was due to the -CH_2_ scissoring modes (Myrzakozha et al., 1999a; Myrzakozha et al., 1999b). The characteristic peaks of water were observed at 3,439 and 1,630 cm^−1^. The above results illuminated that ODA_10_[(PW_11_Fe)_2_] was of dimeric Keggin-type structure and prepared by electrostatic interaction between quaternary ammonium cations and heteropoly anions.

UV-vis DRS of the dimeric ODA_10_[(PW_11_Fe)_2_] is shown in Figure 1C. The absorption at 260 nm was due to O→W charge transfer transition. The bands at 349 nm and 412 nm corresponded to O→Fe charge transfer transition of oxo-bridged di-iron complexes (Kurtz, 1990). Another evidence of Fe-O-Fe bond presented the characteristic absorptions at 466, 530 and 621 nm which were attributed to O→Fe charge transfer transitions (Kurtz, 1990). So, the IR and UV-vis DRS results all indicated that ODA_10_[(PW_11_Fe)_2_] possessed dimeric structure.

The SEM image of the dimeric ODA_10_[(PW_11_Fe)_2_] is shown in Figure 1D. The particles of ODA_10_[(PW_11_Fe)_2_] were slightly irregular in shape and the particle size was about 510 nm measured by DLS (Figure 1E), which was consistent with the result of SEM image. The obtained particles showed amorphous morphology, which might be caused by the relatively long carbon chain of organic countercations, making no crystalline feature observed in ODA_10_[(PW_11_Fe)_2_], which was also proved by the results of XRD pattern of ODA_10_[(PW_11_Fe)_2_] (Figure 1F). The spectrum recorded for ODA_10_[(PW_11_Fe)_2_] indicated an amorphous feature due to the lack of crystallinity caused by ODAB, which had strong diffraction peaks at the 2θ degree of 6.9^°^–10.3^°^ and a weak broad peak at 14.3^°^–39.2^°^. The characteristic diffractions of Keggin-type polyoxometalate were detected at 8.28^°^, 8.9^°^, 9.1^°^, 27.9^°^ and 28.9^°^, etc (Jalil et al., 2003), so it is concluded that ODA_10_[(PW_11_Fe)_2_] was of Keggin-type structure. The broad peak at 14.3^°^–39.2^°^ manifested again that the poor crystallinity of ODA_10_[(PW_11_Fe)_2_] resulted from the longer carbon chain of quaternary ammonium cations. The above results of SEM and XRD proved that the dimeric ODA_10_[(PW_11_Fe)_2_] had amorphous morphology and contained Keggin-type structure after quaternary ammonium cations combining with heteropoly anions.

### Anticancer Activity Studies

**FIGURE 2:**
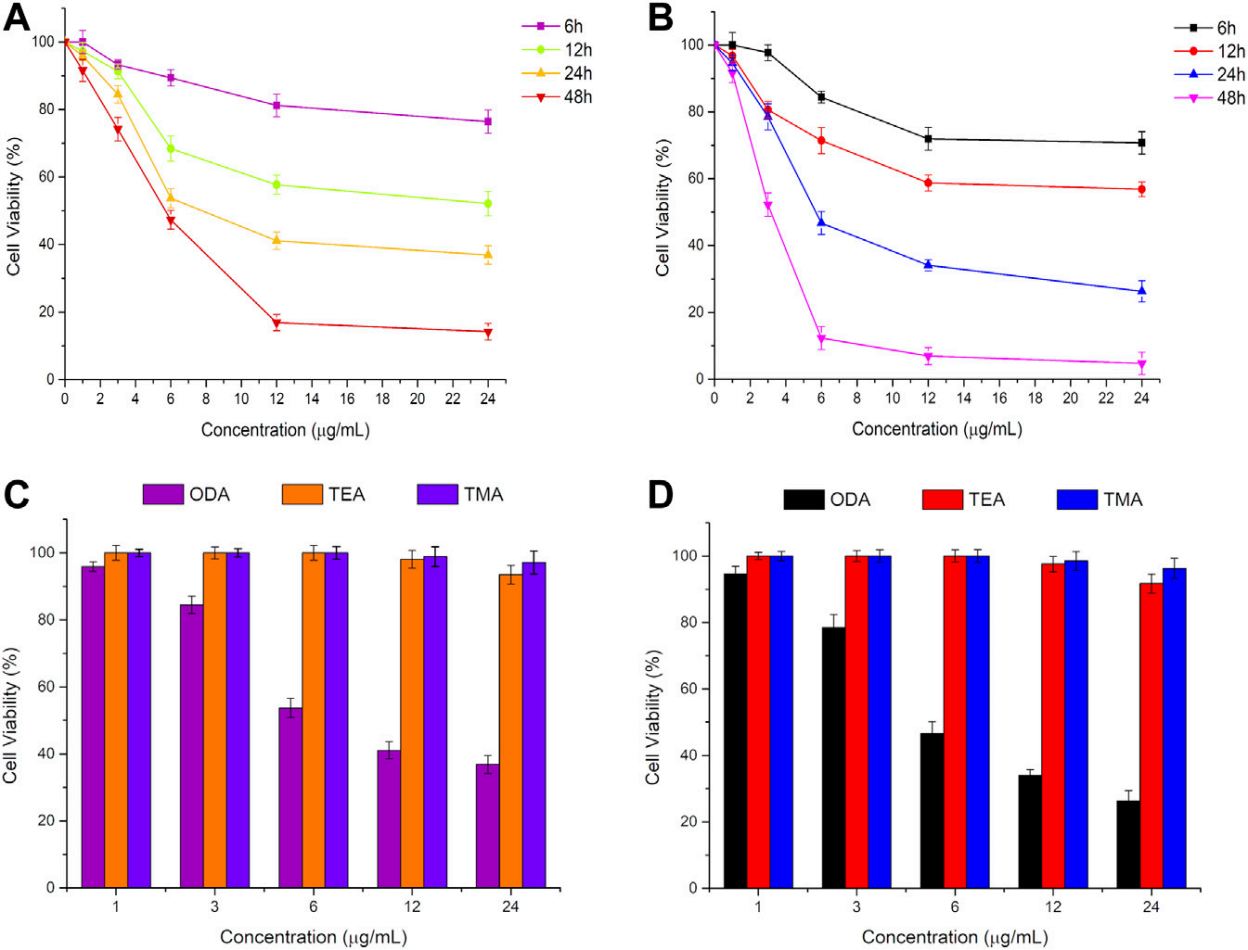
*In vitro* cytotoxicity profiles of ODA_10_[(PW_11_Fe)_2_] on MCF-7 cells **(A)** and A549 cells **(B)** for 6, 12, 24 and 48 h, and different phosphotungstates on MCF-7 cells **(C)** and A549 cells **(D)** for 24 h by MTT at different doses. (ODA: ODA_10_[(PW_11_Fe)_2_]; TEA: (TEA)_10_[(PW_11_FeO_39_)_2_O]·3H_2_O; TMA: (TMA)_10_[(PW_11_FeO_39_)_2_O]·4H_2_O).

The *in vitro* anti-proliferation activity of ODA_10_[(PW_11_Fe)_2_] on MCF-7 and A549 cells was evaluated by the MTT assay. Cells were exposed to different concentrations of ODA_10_[(PW_11_Fe)_2_] (1, 3, 6, 12, 24 μg/ml) for 6, 12, 24 and 48 h. As shown in Figures 2A,B, the cell viability decreased with the concentration of ODA_10_[(PW_11_Fe)_2_] increasing, which illuminated that the anti-proliferative effects of ODA_10_[(PW_11_Fe)_2_] depended on its concentration. For MCF-7 cells, after treatment with ODA_10_[(PW_11_Fe)_2_], the cell viability declined to 76.4 at 6, 52.1 at 12, 36.9 at 24 and 14.2% at 48 h. Analogously, for A549 cells, the cell viability was 70.7, 56.8, 26.3 and 4.7% at 6, 12, 24 and 48 h, respectively. The above results indicated that ODA_10_[(PW_11_Fe)_2_] exhibited inhibitory activity against tumor cells growth in a time-dependent effect during these periods. IC_50_ values of ODA_10_[(PW_11_Fe)_2_] against MCF-7 and A549 cells at different time were presented in Table 1. The calculated IC_50_ values in MCF-7 cells for 24 and 48 h were 8.02 μg/ml and 5.83 μg/ml, respectively, and for A549 cells, the IC_50_ value was observed to be 5.75 μg/ml at 24 h and 3.23 μg/ml at 48 h. For the short treatment time groups, 6 h and 12 h, of MCF-7 and A549 cells, the inhibition rate was lower than 50%, thus IC_50_ values in MCF-7 and A549 cells for 6 h and 12 h were not obtained in Table 1. Nevertheless, IC_25_ values of ODA_10_[(PW_11_Fe)_2_] against MCF-7 and A549 cells at different times were calculated and shown in Table 1. For MCF-7 cells, IC_25_ values were 29.28 μg/ml, 5.05 μg/ml, 4.13 μg/ml and 2.78 μg/ml at 6, 12, 24 and 48 h, respectively, and IC_25_ values in A549 cells for 6, 12, 24 and 48 h were 10.68 μg/ml, 4.71 μg/ml, 3.35 μg/ml and 1.92 μg/ml, respectively. The IC_50_ and IC_25_ values were sharply reduced when the drug treatment time prolonged. These data suggested that ODA_10_[(PW_11_Fe)_2_] could inhibit MCF-7 and A549 cells growth in a time-dependent manner, and the antiproliferation effect on A549 cells was stronger than that on MCF-7 cells.

**TABLE 1:** IC_50_ and IC_25_ values of ODA_10_[(PW_11_Fe)_2_] against MCF-7 and A549 cells.

Cell line	IC_50_ (μg/ml)				IC_25_ (μg/ml)			
	6 h	12 h	24 h	48 h	6 h	12 h	24 h	48 h
MCF-7	—	—	8.02	5.83	29.28	5.05	4.13	2.78
A549	—	—	5.75	3.23	10.68	4.71	3.35	1.92

Moreover, the antitumor activity of ODA_10_[(PW_11_Fe)_2_] was compared with that of dimeric (TEA)_10_[(PW_11_FeO_39_)_2_O]·3H_2_O (abbreviated as TEA) and (TMA)_10_[(PW_11_FeO_39_)_2_O]·4H_2_O (abbreviated as TMA) containing relatively short alkyl chain at the same concentration, which was shown in Figures 2C,D. After treatment of the drugs for 24 h, ODA_10_[(PW_11_Fe)_2_] showed the highest anticancer effect, while nearly no inhibition efficacy against MCF-7 and A549 cells was induced by TEA and TMA, that is because the longer alkyl chain in the quaternary ammonium cation of ODA_10_[(PW_11_Fe)_2_] possessed better cell membrane penetration (Abel et al., 2002), which is beneficial for ODA_10_[(PW_11_Fe)_2_] to interact with tumor cells, thus ODA_10_[(PW_11_Fe)_2_] exhibited excellent antiproliferation effect on MCF-7 and A549 cells. The above results demonstrated that the dimeric Keggin-type POMs modified by quaternary ammonium cation with longer alkyl chain showed higher antitumor activity.

**FIGURE 3:**
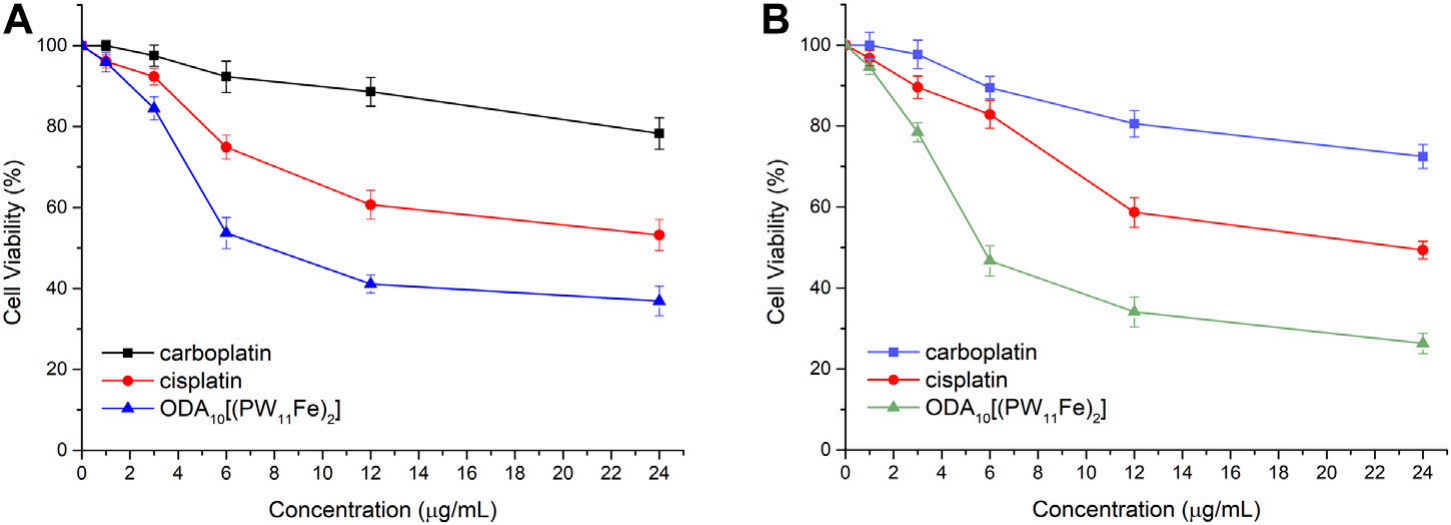
*In vitro* cytotoxicity profiles of different drugs on MCF-7 cells **(A)** and A549 cells **(B)** for 24 h.

The anticancer activity of ODA_10_[(PW_11_Fe)_2_] on MCF-7 and A549 cells was further compared with that of the clinical chemotherapeutic agents, such as cisplatin and carboplatin, under the same conditions, which was presented in Figure 3. After treatment of the drugs for 24 h, ODA_10_[(PW_11_Fe)_2_] showed the strongest inhibitory effects against MCF-7 and A549 cells compared to cisplatin and carboplatin. From the above results, it can be demonstrated that ODA_10_[(PW_11_Fe)_2_] exhibited an anti-proliferation effect on the tumor cells in a dose- and time-dependent manner, and can be utilized as an antitumor drug candidate for the treatment of cancer.

### Cell Morphology

**FIGURE 4:**
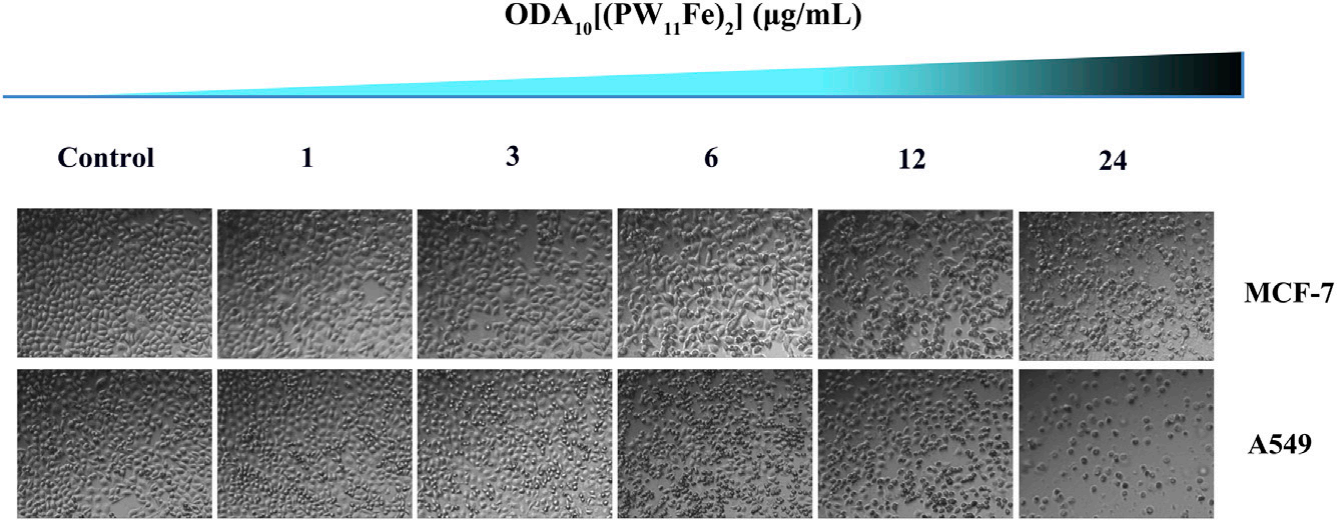
Morphological changes of MCF-7 and A549 cells induced by different concentration of ODA_10_[(PW_11_Fe)_2_] for 24 h.

The changes of the cellular density and morphology of MCF-7 and A549 cells with the treatment of different doses of ODA_10_[(PW_11_Fe)_2_] were directly detected using an inverted microscope (Figure 4). After treatment of 24 h, cells of control group were nested distribution, flattened and showed normal cell architecture. The cell shape was regular polygon and few round cells existed. While the morphology of the ODA_10_[(PW_11_Fe)_2_]-treated cells obviously changed. The cells were becoming round, shrunken, altered adherence, as well as the appearance of a large number of cell membrane blistering, which was the characteristic morphology of apoptosis. Moreover, the density of the cells was reduced with the drug concentration increasing, showing a dose-dependent effect. This phenomenon indicated that ODA_10_[(PW_11_Fe)_2_] could induce cell apoptosis.

### Flow Cytometry Analysis of Cell Apoptosis

**FIGURE 5:**
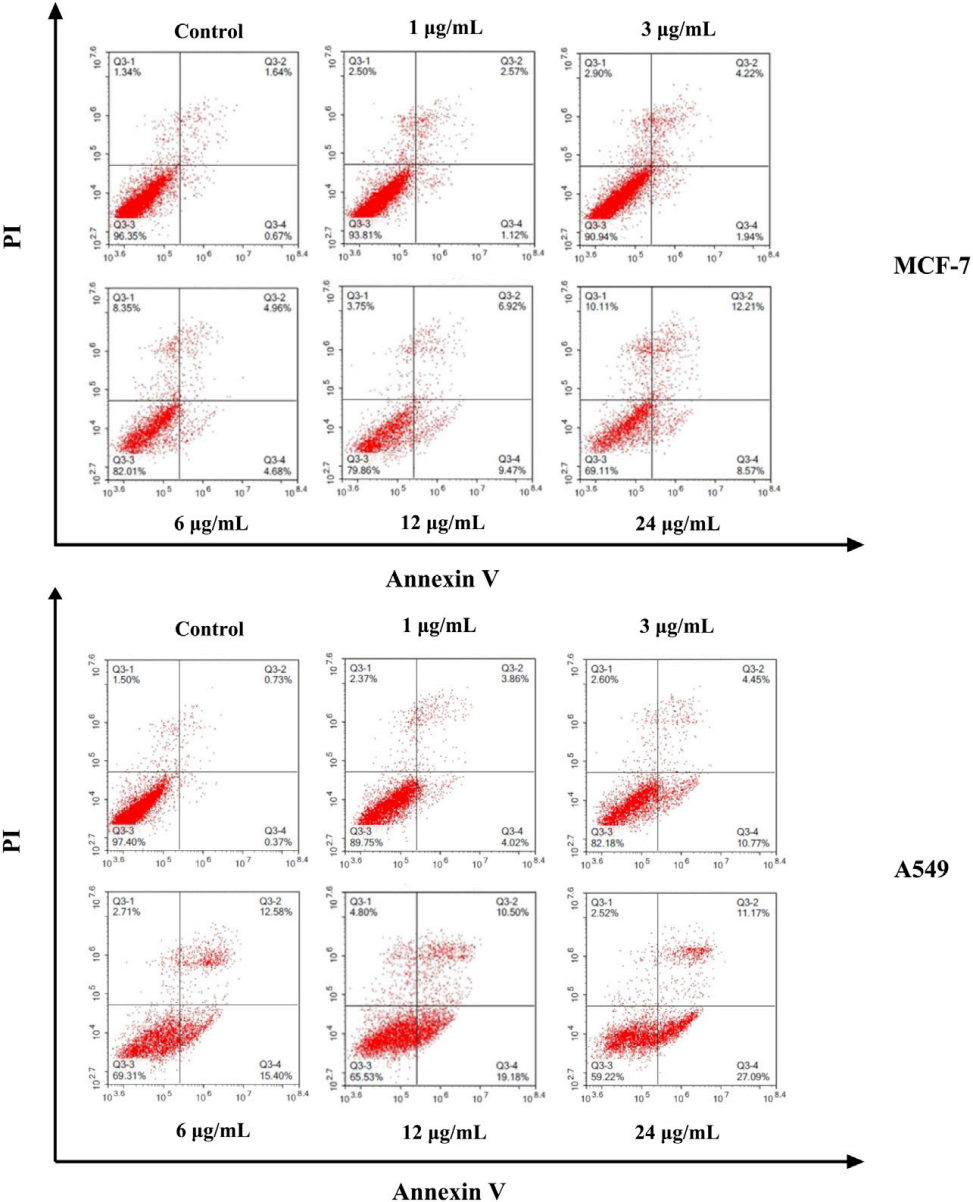
Flow cytometry analysis of MCF-7 and A549 cell apoptosis induced by different concentration of ODA_10_[(PW_11_Fe)_2_] for 24 h.

The cell apoptosis against MCF-7 and A549 cells induced by different concentrations of ODA_10_[(PW_11_Fe)_2_] (1, 3, 6, 12, 24 μg/ml) for 24 h was evaluated using Annexin V-FITC/PI double-staining technique. As shown in Figure 5, the apoptotic cells could be obviously detected by distinct double staining patterns: necrotic (upper left square), viable cells (lower left square), late apoptotic (upper right square) and early apoptotic (lower right square). The results manifested that the ratio of apoptotic (early and late) cells induced by ODA_10_[(PW_11_Fe)_2_] obviously increased in a dose-dependent manner compared to control group. For MCF-7 cells, after treated with ODA_10_[(PW_11_Fe)_2_], the proportion of apoptotic cells ranged from 3.69 to 20.78% with the concentration increasing, which was higher than that in control group (2.31%). For A549 cells, the percentage of apoptotic cells in control group was 1.1%, while that in drug-treatment group was 7.88, 15.22, 27.98, 29.68 and 38.26% at the concentration of 1–24 μg/ml, respectively. Taken together, ODA_10_[(PW_11_Fe)_2_] could inhibit the MCF-7 and A549 cells growth and induce the apoptosis of tumor cells.

### Flow Cytometry Analysis of Cell Cycle Distribution

In order to detect whether the antiproliferation effect against MCF-7 and A549 cells of ODA_10_[(PW_11_Fe)_2_] is caused by cell cycle arrest, MCF-7 and A549 cells were treated with different doses of ODA_10_[(PW_11_Fe)_2_] (1, 3, 6, 12, 24 μg/ml) for 24 h with PI staining. The content of DNA was examined by flow cytometry. Generally, the process of cell replication is related to the doubling of DNA and other cellular contents. There are four distinct phases divided from cell cycle distribution: G_1_, S, G_2_ and M phase, the entry to which is carefully regulated by different checkpoints. S phase is responsible for the synthesis of DNA. The cell prepares to divide during G_2_ phase and division takes place during M phase. Then, the cells continue to divide after passing these checkpoints. Moreover, many external factors such as drugs, radiation and ROS (reactive oxygen species) can induce DNA-damage during S which causes the death of cells (Rodriguez-Vargas et al., 2012; Preya et al., 2017).

**FIGURE 6:**
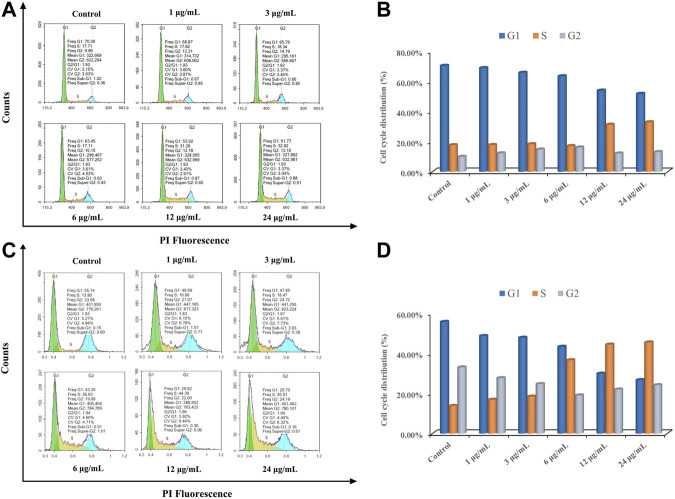
ODA_10_[(PW_11_Fe)_2_] induces cell cycle arrest in MCF-7 and A549 cells. MCF-7 cells **(A)** and A549 cells **(C)** were treated with various concentrations of ODA_10_[(PW_11_Fe)_2_] for 24 h, and cell cycle arrest was examined by flow cytometry. Quantitative bar graphs of the proportion of MCF-7 cells **(B)** and A549 cells **(D)** in different phases.

The results of cell cycle arrest of MCF-7 and A549 cells induced by different concentrations of ODA_10_[(PW_11_Fe)_2_] from 1 to 24 μg/ml are shown in Figure 6. The cell population at S phase of MCF-7 cells in the drug-treatment group increased from 17.82 to 32.92%, in a dose-dependent effect, which was higher than that in control group (17.71%). The level of G_2_/M phase exhibited no obvious variations after treatment of ODA_10_[(PW_11_Fe)_2_], accompanied by a significant reduction in G_1_ phase (Figure 6A). Similar to MCF-7 cells, as shown in Figure 6B, for A549 cells, the level of S phase increased from 13.8% in the control group to 16.89, 18.47, 36.63, 44.39, and 45.51%, respectively, with the proportions of G_1_ and G_2_/M phase decreasing. The ratios of G_1_ and G_2_/M phase decreased from 55.74 to 26.7% and 33.08 to 24.19%, respectively. Since DNA replicates during S phase, the above results manifested that DNA damaged at S phase and the antitumor mechanism on MCF-7 and A549 cells was S phase arrest.

## Conclusion

A novel dimeric Keggin-type polyoxometalate (ODA)_10_[(PW_11_FeO_39_)_2_O]·9H_2_O (ODA_10_[(PW_11_Fe)_2_]) was firstly synthesized with the aid of octadecyltrimethylammonium cation. A comprehensive study on antitumor activity of ODA_10_[(PW_11_Fe)_2_] against MCF-7 and A549 cells was carried out. ODA_10_[(PW_11_Fe)_2_] could inhibit MCF-7 and A549 cells growth in a dose- and time-dependent manner, and the IC_50_ value for MCF-7 and A549 cells was 5.83 μg/ml and 3.23 μg/ml at 48 h, respectively. The higher antitumor activity was due to the better cell membrane penetration of octadecyltrimethylammonium cation with longer alkyl chain. The morphology of MCF-7 and A549 cells treated with ODA_10_[(PW_11_Fe)_2_] exhibited the characteristics of apoptosis. The Flow cytometry analysis results manifested the fact of the cell apoptosis and cycle arrested at S phase induced by ODA_10_[(PW_11_Fe)_2_] as the main mechanism for antiproliferation of MCF-7 and A549 cells. Our work has demonstrated that ODA_10_[(PW_11_Fe)_2_] can be utilized as an antitumor drug candidate for the treatment of cancer.

## Data Availability Statement

The original contributions presented in the study are included in the article/Supplementary Material, further inquiries can be directed to the corresponding author.

## Author Contributions

YX and WL designed the experiments. YY, MC, and JG carried out the experiments and wrote the manuscript. GC and HL helped analyzing the experimental results.

## Funding

This work was financially supported by the Grant of Jilin Province Science & Technology Committee (No. 20180101194JC).

## Conflict of Interest

Author HL was employed by the company NCPC Hebei Lexin Pharmaceutical Co., Ltd.

The remaining authors declare that the research was conducted in the absence of any commercial or financial relationships that could be construed as a potential conflict of interest.
